# Effects of Post-Exercise Heat Exposure on Acute Recovery and Training-Induced Performance Adaptations: A Systematic Review

**DOI:** 10.1186/s40798-025-00910-0

**Published:** 2025-10-01

**Authors:** Essi K. Ahokas, Richard S. Hennessy, Helen G. Hanstock, Heikki Kyröläinen, Johanna K. Ihalainen

**Affiliations:** 1 Biology of Physical Activity, Faculty of Sport and Health Sciences, University of Jyväskylä, Jyväskylä, Finland; 2 Swedish Winter Sports Research Centre, Department of Health Sciences, Mid Sweden University, Östersund, Sweden; 3 Finnish Institute of High Performance Sport KIHU, Jyväskylä, Finland

**Keywords:** Sauna, Hot water immersion, Endurance performance, Neuromuscular performance, Recovery, Adaptations, Heat acclimation

## Abstract

**Background:**

Whole-body heat exposure, such as sauna bathing or hot water immersion (HWI) has been shown to induce various physiological adaptations that can improve athletic performance. However, the effects of post-exercise heat exposure on acute recovery and promoting training-induced performance adaptations are not well understood. The aim of this systematic review was to summarise the current evidence on the effects of post-exercise heat exposure on physical performance in healthy adults.

**Methods:**

This review followed the Preferred Reporting Items for Systematic Reviews and Meta-Analyses (PRISMA) guidelines. A systematic search for articles was conducted in December 2023 and updated in June 2025 using the PubMed, SPOLIT, Medline, and SPORTDiscus databases. Eligible studies were randomised or crossover trials comparing whole-body post-exercise heat exposure (≥ 36 °C, e.g., hot water immersion to at least sternum level, or sauna bathing) to passive or placebo recovery. The risk of bias of the included studies was assessed using the Cochrane Collaboration Risk of Bias Tool version 2. Only studies that provided results on maximal physical performance outcomes in healthy adults were included.

**Results:**

Fourteen studies, including a total of 194 participants, met the inclusion criteria. Nine studies investigated acute effects after heat exposure and five were long-term training intervention studies. The acute studies reported uncertain results, with studies showing no effects (*n* = 4), beneficial (*n* = 4), or adverse (*n* = 1) effects of post-exercise heat exposure on performance recovery. The chronic studies suggested that post-exercise heating may improve running performance, at least in hot conditions. However, repeated heat exposures had no effect on cycling performance or VO_2_max. The overall quality of the evidence was low to moderate. The heterogeneity of study designs, heating protocols, exercise modes, performance outcomes and recovery times precluded meta-analysis.

**Conclusion:**

Based on the current evidence, it is not possible to draw definitive conclusions about the effects of post-exercise heat exposure on recovery and physical performance development. Additional high-quality studies are needed to determine the optimal heat exposure methods and recovery strategies for different types of training and performance outcomes.

## Key Points

The effects of post-exercise whole-body heating on acute recovery of physical performance are not clear, although some improvements in physical performance have been observed immediately after heat exposure.

Regular use of post-exercise heating methods may improve endurance performance if physiological adaptations related to heat acclimation also occur.

Future studies should focus on the effects of repeated use of post-exercise heating on training adaptations of power and strength capacities.

Physiological responses during heat exposure should also be reported in future studies since both the methods employed and the research findings are so diverse.

## Background

Heating interventions have been widely used for body-mass reduction in combat sports [1]. However, whole-body heating methods, such as saunas, heated chambers, and hot water immersion (HWI), have also gained popularity in endurance and team sports [2, 3]. A survey study across several sports revealed that 62% of athletes (*n* = 295) used heating strategies in their training [2]. Alongside weight-control, heat exposures have been used for recovery, rehabilitation, acclimation, and warming-up purposes [2]. Furthermore, interest in the use of post-exercise saunas and HWI for passive heat acclimation has been observed, as they may be more accessible and time efficient strategies than exercise-based approaches [4].

The impact of saunas [5] and HWI [6] on acute recovery of performance and adaptations to training appear equivocal and contradictory, which may be explained by differences in methodologies, including exercise sessions or training interventions, outcome measures of performance, types of recovery strategies applied, and participant characteristics. There is some evidence that long-term heating without physical training may enhance muscle hypertrophy [7] and maximal force production [7–9] in sedentary participants, but these results may not be generalisable to trained populations due to their pre-existing exercise-induced adaptations [10].

The primary of this study was to investigate the effects of whole-body heat exposure interventions on the acute recovery of physical performance following a single training session or a brief training period. The second aim was to study the effects of the repeated use of heat exposure in combination with training on the development of physical performance.

## Methods

This systematic review was performed according to the Preferred Reporting Items for Systematic Reviews and Meta-Analysis (PRISMA) and was not pre-registered.

### Data Sources and Searches

A literature search was conducted (EKA) of the PubMed (MEDLINE), SPOLIT (SportLiteraturdatenbank), MEDLINE (Ovid), and SPORTDiscus databases. To identify the articles, Boolean operators were created where passive heating was combined with physical performance: ((sauna, infrared sauna, infrared radiation, heated chamber, steam room, heat-therapy, OR hot water immersion) AND (recover*, training adaptation*, physical performance, neuromuscular performance, performance, strength, endurance, OR body composition) NOT (rats OR mice)).

The literature search was performed in December 2023, and again in June 2025. The latter search included only the years 2023–2025. Only English-language and peer-reviewed studies were searched. Inclusion and exclusion criteria are presented in Table 1.

**Table 1:** Inclusion and exclusion criteria based on PICOS (Population, intervention, control, outcome, and study design)

	Inclusion	Exclusion
**P**	Age 15–60 years	Age < 15 and > 60 years, Overt chronic diseases
**I**	Post-exercise heat methods, Air or water temperature > 36 °C, HWI sternum level or higher, Include training session / intervention	Pre-exercise or during exercise methods, Air or water temperature < 36 °C, No external source of heat, Only local heat treatment (under the sternum level), Not include training session / intervention, The training differed between groups / interventions
**C**	Passive recovery / training without recovery methods / placebo, Or TWI in HWI	Compared only to other recovery methods
**O**	Maximal physical performance variables	Not measured maximal physical performance
**S**	Randomised controlled trials, Crossover design	Not randomised controlled trials, Crossover design

*HWI* hot water immersion, *TWI* thermoneutral water immersion

### Study Selection

Following the removal of duplicate results, two researchers (EKA and RH) independently screened the titles and abstracts. The same researchers then examined the eligibility of the full-text papers. Disagreements during the screening and eligibility assessment were resolved by a third researcher (JKI).

### Data Extraction

The following data from the included studies were extracted by one researcher (EKA): study design (randomised trial or crossover design), participant characteristics (sample size, age, sport, training level), characteristics of the interventions (timing, durations, temperature, and frequency of recovery methods and control; training protocol/intervention), test protocols (tested measures, timing), and results (between-groups and within-groups). Included studies were divided into (1) acute recovery studies, and (2) training adaptation and long-term effect studies.

### Risk of Bias Assessment

The risk of bias of included studies was assessed using the Cochrane Collaboration Risk of Bias Tool version 2 for randomised trials and Risk of Bias Tool version 2 for crossover trials [11]. Risk of bias was evaluated according to the domains (i) randomisation process; (ii) deviations from intended interventions; (iii) missing outcome data; (iv) measurement of the outcome and (v) selection of the reported result. In crossover trials, the sixth domain (s) period and carryover effects were evaluated. Two researchers (EKA and RH) independently assessed studies for risk of bias. Any disagreements were resolved by discussions between three researchers (EKA, RH, and JKI).

## Results

### Study Selection

**Fig. 1:**
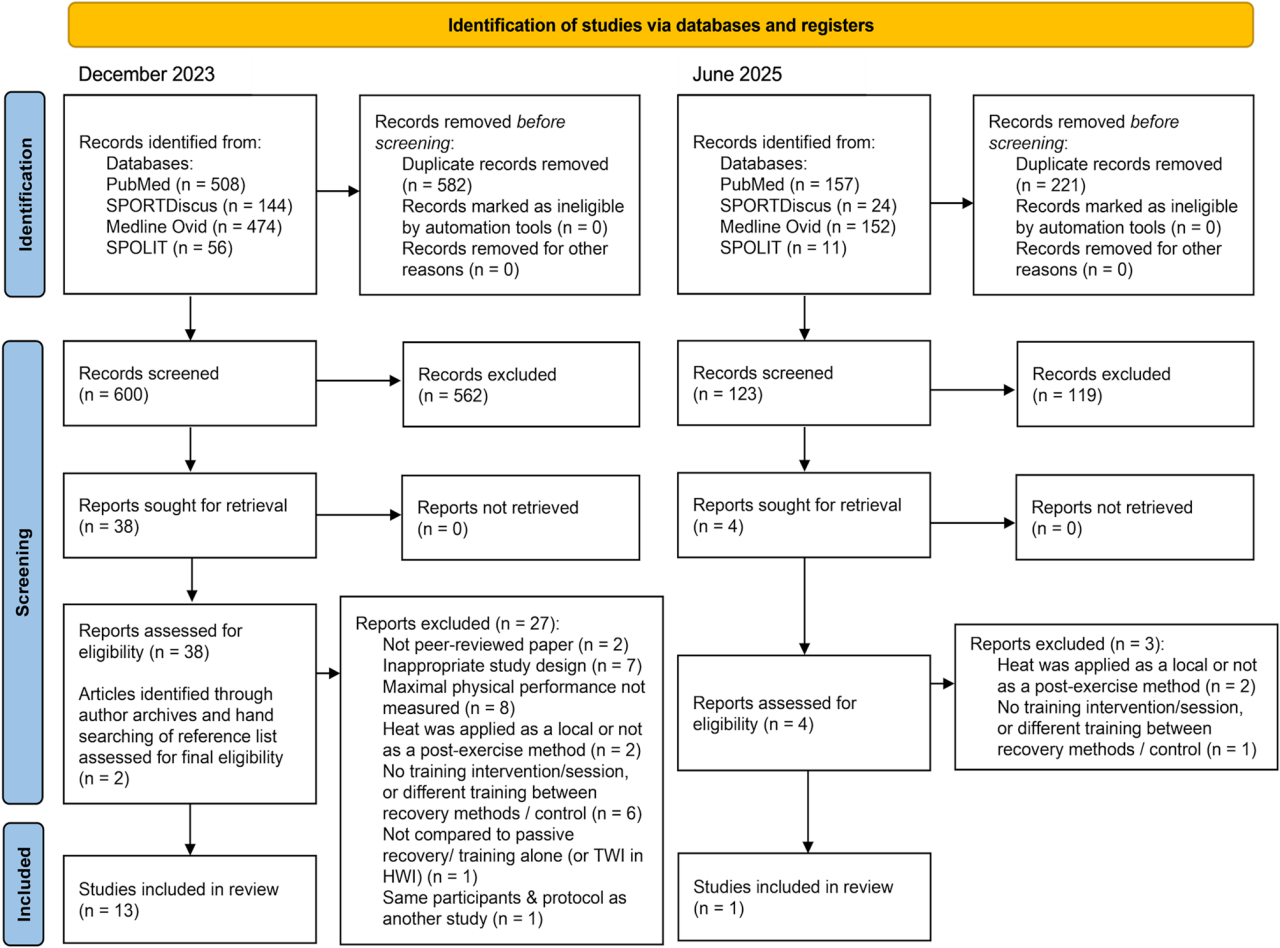
Flow chart illustrating the literature search and selection of studies. *HWI* hot water immersion, *TWI* thermoneutral water immersion

Of 1182 records identified from the literature search in December 2023, 582 duplicates were removed, and 600 records were screened, of which 562 records were excluded based on titles and abstracts. The remaining 38 records from the literature search were added to two records identified by the authors from archives and reference lists. These 40 records were then screened in full. In June 2025, 344 records were identified, of which 221 duplicates were removed, and 123 titles and abstracts were screened. Of these, the full text of four records was screened. Finally, fourteen studies were included in the review (Fig. 1).

### Study Characteristics

Of the 14 studies included, 3 were randomised controlled trials [12–14] and 11 were crossover trials [10, 15–24]. A total of nine studies investigated acute recovery and five studies investigated training adaptations and long-term effects on physical performance. Of the acute recovery studies, six used HWI as a post-exercise recovery method, whilst traditional sauna was used in one study and infrared sauna (IRS) in two studies. In training adaptation studies, three used traditional saunas and two used HWI as a post-exercise recovery method. The total number of participants in the systematic review was 194 and the number of participants ranged from 6 to 20 per intervention or control group. Three studies had < 10 participants per group [17, 18, 20]. Participants’ level of physical activity ranged from recreationally active to elite athletes. Only male participants were recruited in 11 studies, only female participants were recruited in 1 study, and both female and male participants were included in 2 studies [18, 22].

### Heat Exposure Effects on Acute Recovery of Physical Performance

Studies investigating the effects of heat-based recovery methods on acute recovery of physical performance are presented in Table 2. Four studies investigated recovery of endurance performance. Recovery of power and sprint performance was examined in five studies and isometric maximal force production in five studies.

**Table 2:** Studies investigating acute recovery of physical performance

Study		Participant	Recovery methods			Methods				Results	
	**Design**	**N (sex), participant classification (tier)** [25]**, training background, age**	**Method (temperature; duration; body position)**	**Number of protocols; timing**	**Control / passive recovery (temperature**,** duration)**	**Exercise loading protocol**	**Test protocols**	**Test variable**	**Time-points**	**Group and interaction effects**	**Time effects**
De Paula et al. 2018 [17]	Cross-over design	9 (M) Tier 2 recreationally trained 24 (6) years	HWI (38 °C; 15 min; seated submerged to the sternum level)	Once Immediately post-ex	PAS (Room temperature; 15 min; seated)	Eccentric unilateral knee flexion 3 × 10 & Run: 2 × 45 min at 70% of VO_2peak_	5 km time-trial (running)	Average speed	4.5 h post-rec	No differences between groups, *p* > 0.05	
								Average speed of each kilometer		No differences between groups, *p* > 0.05	
Vaile et al. 2008 [24]	Cross-over design	12 (M) Tier 3 cyclists 32.2 (4.3) years	HWI (38 °C; 14 min; submerged to the neck)	5 consecutive days Immediately post-ex	PAS (14 min)	Cycle protocol on 5 consecutive days. Totalled approx. 105-min in duration, consisting of 66 maximal effort sprints (5–15 s) and total of 9-min of sustained effort.	Sprint performance	Average power	Day 1–5	*p* > 0.05	HWI: decreased 0.6–3.7%, PAS: decreased 1.7–4.9%
							Time trial performance	Total work	Day 1–5	*p* > 0.05	
								Average power		**Day 3: PAS lower vs. HWI: *p* = 0.02**, other days *p* > 0.05	HWI: an improvement of 1.5% to a decrease of 3.4%, PAS: decreased 2.6–3.8%
Coertjens et al. 2023 [16]	Cross-over design	21 (M) Tier 3 cyclists and triathletes 26.9 (5.8) years	HWI (40 °C; 10 min; seated submerged to the sternum level)	Once Immediately post-ex	PAS (Room temperature; 10 min; seated)	Wingate test on a cycle ergometer	A second Wingate test	Mechanical work	Pre & immediately post-rec	HWI vs. CON: *p* > 0.05	
								Mean power output		**Higher after HWI vs. CON: ** ***p*** ** < 0.05**	**Post 3.2% higher than pre in HWI (** ***p*** ** < 0.001)**
								Peak power output		HWI vs. CON: *p* > 0.05	
								Mean distance		**Higher after HWI vs. CON: ** ***p*** ** < 0.05**	**Post higher than pre in HWI (** ***p*** ** < 0.001)**
Vaile et al. 2008 [23]	Cross-over design	11 (M) Tier 2 strength trained	HWI (38 °C; 14 min; submerged to the neck)	4 x during the 72 h recovery period Immediately post-ex, at 24, 48, and 72 h post-ex	PAS (14 min)	5 × 10 eccentric bilateral leg press with a load of 120% of 1-RM followed by 2 × 10 at a load of 100% 1-RM.	Strength performance	Isometric squat	Pre & 0, 24, 48, and 72 h post-exercise	**Δ%: reduced less following HWI (*p* < 0.05) vs. PAS at post24, 48**,** and 72 h**	
								Loaded squat jump		HWI vs. PAS: *p* > 0.05	**HWI & CON at post72h: reduced vs. pre: ** ***p*** ** < 0.05**
Wellauer et al. 2025 [14]	RCT	HWI: 10 (F), 23.1 (3.6) years CON: 10 (F), 23.1 (1.6) years Tier 2 recreationally active	HWI (40 (0.5)°C, submerged to sternum level, 2 × 10 min)	Twice Immediately post-exercise and 2 h after	CON (Room temperature 21 (2) °C and 40 (5) % RH, 2 × 10 min)	5 × 20 drop-jumps (0.6 m box)	Isometric strength of right knee extensor	MVC	Pre & 24 h, 48 h, and 72 h post-exercise	HWI vs. CON *p* > 0.05	**Time effect**, ***p* < 0.001**
Horgan et al. 2023 [10]	Cross-over design	18 (M) Tier 3 volleyball players 19.9 (3.4) years	HWI (39.1 (0.5) ◦C; 15 min; submerged to the neck)	Once 30 min post-ex	PAS (Room temperature 23.7 (0.4) ◦C; 15 min)	Hypertrophic resistance training session: 8 exercises, 3–5 sets for 10 repetitions at 75% of the 1RM	Isometric midthigh pull	Absolute peak force	Pre & 0, 1, 3, 14, and 38 h post-exercise	No treatment x time effects, *p* > 0.05	**Time effect**, ***p* = 0.007**
								Absolute time-specific force at 200 ms and avarage RFD		No treatment x time effects, *p* > 0.05	**Time effect**, ***p* < 0.001**
							Jump tests	SJ peak power		Interaction effects, *p* > 0.05	**Time effect**, ***p* < 0.001**
								SJ height & RFD		Interaction effects, *p* > 0.05	Time effect, *p* > 0.05
								CMJ peak power		**Treatment effect: HWI higher vs. CON *p* = 0.002 (g = 0.14)**; Interaction effects, *p* = 0.728	**Time effect**, ***p* < 0.001**
								CMJ height		**Treatment effect: HWI higher vs. CON *p* = 0.017 (g = 0.19)**; Interaction effects, *p* = 0.354	**Time effect**, ***p* < 0.001**
								CMJ RFD		**Treatment effect: HWI higher vs. CON *p* = 0.037 (g = 0.34)**; Interaction effects, *p* = 0.153	Time effect, *p* > 0.05
Skorski et al. 2019 [22]	Cross-over design	20 (F: 3, M: 17) Tier 3–4 triathletes and swimmers 17.3 (2.1) years	Sauna (80–85 °C and 10% RH; 3 × 8 min; sit in an upright/lay in a supine position)	Once	Placebo (applied massage oil to their body while passively resting; room temperature: ∼28 °C; 35 min)	Swimming: technical drills and 50- to 100-m intervals, resulting in a total volume of 5000 m.	4 × 50 m all-out sprint	Overall time	Pre and post (following morning) exercise	**Interaction effect ** ***p*** ** = 0.02; η2p = 0.24**	SAU: increased 1.69% (95% CI: -0.76–0.48); PLAC: decreased 0.66% (95% CI: -0.57–0.66)
								Split times		**Slower in the first 50 m after SAU vs. PLAC (interaction effect *p* = 0.03)**; Other split times: interaction effect *p* > 0.05	Time effect: *p* > 0.05
							CMJ	Average of 4 jumps	Pre and post (following morning)	Interaction effect *p* = 0.35; (ES: η2p = 0.35)	
Ahokas et al. 2023 [15]	Cross-over design	16 (M) Tier 3 basketball players 18.9. (2.3) years	IRS (43 (5) °C at the seat level; RH 21 (1) %; 20 min; sat upright)	Once Immediately post-ex	PAS (Room temperature; 20 min)	1) Back squat 3 × 3 (90–95% 1RM) + CMJ 3 × 3; 2) Nordic hamstring curl 3 × 5 + standing long jump 3 × 3; 3) Leg press 3 × 3 (90–95% 1RM) + box jump 3 × 3.	Physical performance measures	CMJ	Pre & post 14 h	IRS vs. PAS: *p* = 0.129 (ES = 0.65) at post14h; **reduction smaller after IRS vs. PAS: *p* = 0.009 (ES 0.76)**	**PAS decreased pre-post14h: *p* = 0.002 (ES = 1.28)**, IRS *p* > 0.05
								20 m sprint		*p* > 0.05	**IRS & PAS time increased pre-post14h: ** ***p*** ** < 0.001**
								Isometric leg press		*p* > 0.05	IRS & PAS pre-post14h: *p* > 0.05
Mero et al. 2015 [19]	Cross-over design (STS + IRS, STS + CON, ETS + IRS, ETS + CON)	10 (M) Tier 2 recreationally active (PE students) 25.3 (8.4) years	IRS (50 °C at the bather’s face; RH 25–35%; 30 min; sat upright)	Once Post-ex	CON (Room temperature 21 °C; 30 min)	**STS**: 5 × 10RM Leg press and bench press. **ETS**: incremental treadmill run until exhaustion.	Physical performance related to STS	Isometric bench press	Pre & post exercise, & post recovery	IRS vs. CON *p* > 0.05	**IRS & CON: decreased in postEx vs. pre: ** ***p*** ** < 0.001; decreased in postRec vs. pre: ** ***p*** ** < 0.001**
								Isometric leg press		IRS vs. CON *p* > 0.05	**IRS & CON: decreased in postEx vs. pre: ** ***p*** ** < 0.001; decreased in postRec vs. pre: ** ***p*** ** < 0.001**
								CMJ		IRS vs. CON *p* > 0.05	**IRS & CON: decreased in postEx vs. pre: ** ***p*** ** < 0.001; decreased in postRec vs. pre: ** ***p*** ** < 0.001**
							Physical performance related to ETS	CMJ		**IRS higher post-recovery vs. CON: ** ***p*** ** < 0.05**	

*CMJ* counter-movement jump, *CON* control, *ETS* endurance training session, *F* female, *HWI* hot water immersion, *IRS* infrared sauna, *M* male, *PAS* passive recovery, *PE* physical education, *RFD* rate of force development, *RH* relative humidity, *SJ* squat jump, *STS* strength training session, *VO*_*2max*_ maximal oxygen uptake. Bold text indicates *p* < 0.05

Participant classification (tiers) based on McKay et al. [25]: *0* sedentary, *1* recreationally active, *2* trained, *3* national level/highly trained, *4* international level/elite, *5* world class

#### Acute Recovery of Endurance Performance

Recreationally trained males’ recovery of running performance was studied 5 h after exercise loading, which included eccentric strength exercise and a 90 min submaximal running session [17]. No differences in 5 km time-trial average speed were found between HWI (38 °C, 15 min) and passive recovery [17]. Cycling sprint and time-trial performances were investigated on five consecutive days, during which participants underwent daily cycling training sessions along with either HWI at 38 °C for 14 min or passive recovery [24]. No differences in male cyclists’ cycling performance were observed between the two recovery interventions, except for average power in the third day’s time-trial, where HWI resulted in higher values compared to passive recovery [24]. Additionally, in a study by Coertjens et al. [16], male cyclists and triathletes performed a Wingate test followed immediately by either HWI (40 °C, 10 min) or passive recovery. Immediately after the recovery intervention, they performed another Wingate test. Mean power output and mean distance of the second Wingate test were higher after HWI compared to passive recovery. Mechanical work and mean power output did not differ between the recovery interventions [16]. Conversely, all-out swimming performance of young swimmers and triathletes deteriorated after post-exercise sauna bathing (80–85 °C, 3 × 8 min) when the 4 × 50 m all-out swimming test was performed in the morning after the swimming loading, compared to the placebo condition [22]. In particular, the overall time and first split time were slower after the sauna bathing [22].

#### Acute Recovery of Isometric Maximal Force Production

When applied as a recovery intervention after hypertrophic resistance exercise, post-exercise infrared sauna (IRS; 50 °C, 30 min) did not affect subsequent isometric bench press and leg press results of recreationally active men compared to passive recovery, when measured immediately after the IRS exposure [19]. Nor did post-exercise IRS (43 (5) °C, 20 min) improve male basketball players’ recovery of isometric leg press 14 h after a complex resistance exercise session including heavy resistance exercises and jump exercises [15]. Similarly, recovery of male volleyball players’ absolute peak force production during an isometric mid-thigh pull did not differ between HWI (39.1 (0.5) °C, 15 min) and passive recovery during a 38 h recovery period after hypertrophic loading [10]. Additionally, there was no difference between HWI (40 (0.5) °C, 2 × 10 min) and passive recovery in the recovery of knee extensors’ maximal isometric force production of recreationally active women after a drop-jump exercise session [14]. Contradictory results were found in the study of Vaile et al. [23], where declines in peak force during an isometric squat in strength trained men were mitigated by HWI (38 °C, 14 min) compared to passive recovery. In Vaile et al.’s [23] study, HWI was completed four times during the 72-h recovery period and after eccentric resistance exercise loading.

#### Acute Recovery of Power Production and Sprint Performance

IRS exposure was found to improve recovery of counter-movement jump (CMJ) height after complex resistance exercise loading [15] and incremental run loading [19]. However, in the second part of the study [19] involving hypertrophic exercise loading, CMJ performance was not improved by IRS compared to passive recovery. Similarly, IRS did not enhance recovery of 20-m sprint performance in the study [15]. Furthermore, sauna bathing in a traditional sauna did not affect the recovery of CMJ of young swimmers and triathletes compared to passive recovery after swimming loading [22]. HWI did not improve recovery of male volleyball players’ squat jump (SJ) performance after hypertrophic resistance exercise loading [10] or loaded SJ of strength trained men after eccentric resistance exercise loading [23]. However, in the CMJ results, a main effect of treatment was found, showing higher values in the HWI group than in passive recovery, although there were no interaction effects [10].

### Physical Performance Following Training with or Without Repeated Exposure To Heat

Studies investigating the effects of repeated use of heat-based recovery methods on training-induced performance adaptations are summarised in Table 3. Endurance training adaptations were explored in all five studies, while only one study [18] additionally examined training adaptations for speed, maximal isometric strength, and power.

**Table 3:** Studies investigating training adaptations of physical performance

Study		Participant	Recovery methods			Methods				Results	
	**Design**	**N (sex)**,** participant classification (tier)** [25], **training background**,** age**	**Method (temperature; duration; body position)**	**Number of protocols; timing**	**Control / passive recovery (temperature; duration)**	**Training intervention**	**Test protocols**	**Test variable**	**Time-points**	**Group and interaction effects**	**Time effects**
Zurawlew et al. 2016 [13]	RCT	HWI: 10 (M) 23 (3) years CON: 7 (M) 23 (3) years Tier 2 physically active	HWI 39.9 (0.3) °C; 40 min; immersed to the neck	6 times for 6 days	TWI: 34.1 (0.1) °C; 40 min; immersed to the neck	On each day, participants ran for 40 min on a motorised treadmill at 65% VO_2max_	5-km treadmill time trial	Time in 18 °C	Pre & post training period		HWI: *p* > 0.05, TWI: *p* > 0.05
								Time in 33 °C			**HWI: improved (4.9%) *p* = 0.01 (ES: d = 0.42)**, CON: *p* > 0.05
Méline et al. 2021 [18]	Cross-over design	6 (F:3; M:3) Tier 4 short-track speed skaters 21.0 (2.4) years	HWI 40.3 (0.6) °C, RH in the room 92%; 20 min	4 weeks (16 (3) sessions) 10 min after the last training session of the day	PAS (20.3 (0.9), RH about 70%; 20 min)	A generic program after resuming a new season. Similar training loads during both training periods.	Graded exercise test (cycle ergometer)	VO_2max_	Pre & post training period	*p* > 0.05	HWI trend to increase: *p* = 0.053 (ES: d = 0.17), PAS: *p* > 0.05
								Time-to-exhaustion		*p* > 0.05	*p* > 0.05
							Ice tests	1.5-lap all-out time	Pre & post training period	*p* > 0.05	**PAS decreased: *p* = 0.033 (ES: d = 0.21)**, HWI: *p* > 0.05
								1.5-lap all-out: half lap time		*p* > 0.05	**PAS decreased: *p* = 0.049 (ES: d = 0.23)**, HWI: *p* > 0.05
								3- or 7-lap all-out time		*p* > 0.05	*p* > 0.05
							Maximal isometric strength	Knee extensor	Pre & post training period	**HWI higher vs. PAS: ** ***p*** ** < 0.0001**	**PAS decreased: ** ***p*** ** = 0.004 (ES: d = 0.16),** **HWI increased ** ***p*** ** < 0.0001 (ES: d = 0.41)**
								Knee flexor		**HWI higher vs. PAS: ** ***p*** ** = 0.020 (ES: d = 0.20)**	*p* > 0.05
							Jump tests	SJ	Pre & post training period	*p* > 0.05	*p* > 0.05
								CMJ		*p* > 0.05	*p* > 0.05
								Fatigue index		*p* > 0.05	*p* > 0.05
							Force-velocity test (cycling)	P_max_	Pre & post training period	*p* > 0.05	*p* > 0.05
								V_max_		*p* > 0.05	*p* > 0.05
Scoon et al. 2007 [20]	Cross-over design	6 (M) Tier 3 distance runners and triathletes 23 (3) years	Sauna 89.9 (2.0) °C; 31 (5) min; sat upright	3 weeks (totally 12.7 (2.1) sessions); immediately post-exercise	Training without any recovery interventions	Athletes continued their normal training.	Run to exhaustion	Time	Post Sauna-period and post CON-period	**Sauna** ** vs. CON mean (%): 32 (90% CI: (21–43))**	
Karolkiewicz et al. 2022 [12]	RCT	Sauna: 8 (M) CON: 7 (M) Tier 1 PE and physiotherapy students 22.0 (1.5) years	Sauna appr. 90 °C (RH 10 (2) %); weeks 1–2: 3 × 10 min, weeks 3–4: 2 × 15 min	4 weeks (3 times / week)	Training without any recovery interventions	60 min cycle-ergometer 3 x week (weeks 1–2: 50% VO_2peak_, weeks 3–4: 60% VO_2peak_)	Graded exercise test (cycle ergometer)	VO_2peak_	Pre & post training period	Group*time: *p* = 0.15, Group: *p* = 0.584	Time effect: *p* = 0.215
Sitkowski et al. 2022 [21]	Cross-over design	13 (M) Tier 1 PE and physiotherapy students 21.6 (1.6) years	Sauna Head level 105 (7) °C, sternum level 89 (3) °C (RH 10 (2) %); 2 × 15 min; sat upright	4 weeks (3 times per week); almost immediately post-exercise	Training without any recovery interventions	60 min cycle-ergometer 3 x week (week 1–2: 50% Pmax, week 3–4: 60% Pmax)	Graded exercise test (cycle ergometer)	VO_2peak_	Pre & post training periods	Intervention effect: *p* = 0.660; intervention x time: *p* = 0.381	**Time effect (increased): ** ***p*** ** = 0.002 (ES: η2p = 0.323)**
								Maximum power		Intervention effect: *p* = 0.647; intervention x time: *p* = 0.141	**Time effect (increased): ** ***p*** ** < 0.001 (ES: η2p = 0.738)**
								Power at gas exchange threshold		Intervention effect: *p* = 0.729; intervention x time: *p* = 0.252	**time effect (increased): ** ***p*** ** < 0.001 (ES: η2p = 0.634)**

*CMJ* counter-movement jump, *CON* control, *F* female, *HWI* hot water immersion, *M* male, *P*_*max*_ maximum power, *PAS* passive recovery, *PE* physical education, *RCT* randomized controlled trial, *RH* relative humidity, *SJ* squat jump, *TWI* thermoneutral water immersion, *V*_*max*_ maximum velocity, *VO*_*2max*_ maximal oxygen uptake, *VO*_*2peak*_ peak oxygen uptake. Bold text indicates *p* < 0.05

Participant classification (tiers) based on McKay et al. [25]: *0* sedentary, *1* recreationally active, *2* trained, *3* national level/highly trained, *4* international level/elite, *5* world class

#### Heat Exposure Interventions and Running Performance

Six days’ HWI (39.9 (0.3) °C, 40 min/day) following running training (65% of VO_2max_, 40 min/day) improved 5 km time trial performance among recreationally active men in hot conditions (33 °C) [13]. No enhancement was found after thermoneutral water immersion (TWI; 34.1 (0.1) °C), or in temperate conditions (18 °C) in either group [13]. Sub-elite distance runners and triathletes also ran for longer (18.2 (2.0) min vs. 14.1 (2.1) min) in a run-to-exhaustion test after a training period incorporating post-exercise sauna bathing (89.9 (2.0) °C, 31 (5) min, ~ 4 times/week, 3 weeks) compared to training without recovery methods [20]. However, it is noteworthy that participants in the latter study did not undergo pre-tests prior to the training periods.

#### Heat Exposure Interventions and Cycling Performance

Post-exercise sauna bathing (~ 90 °C, 30 min, 3 times/week, 4 weeks) following endurance training [11, 19] and HWI (40.3 (0.6) °C, 20 min, 4 times/week, 4 weeks) following short-track speed skating training [18] did not further improve maximal oxygen uptake (VO_2max_) or peak oxygen uptake (VO_2peak_) measured by a graded exercise test conducted on a cycle ergometer, in comparison to training without any recovery methods. However, there was a trend (*p* = 0.053) toward improved VO_2max_ in speed skaters after training with post-exercise HWI. Improvement over time was not found after training with passive recovery [18].

#### Heat Exposure Interventions and Other Performance Variables

Short-track speed skaters’ three- and seven-lap all-out exercise time did not differ between HWI and passive recovery, nor between pre- and post-tests [18]. However, speed skaters’ 1.5-lap time improved following training with passive recovery, while no change was observed during training with HWI. Conversely, maximal isometric strength of the knee extensor and flexor muscles was greater following HWI compared to passive recovery [18]. There were no significant changes observed in SJ, CMJ, and maximum power and velocity in the force-velocity test [18].

### Risk of Bias

**Fig. 2:**
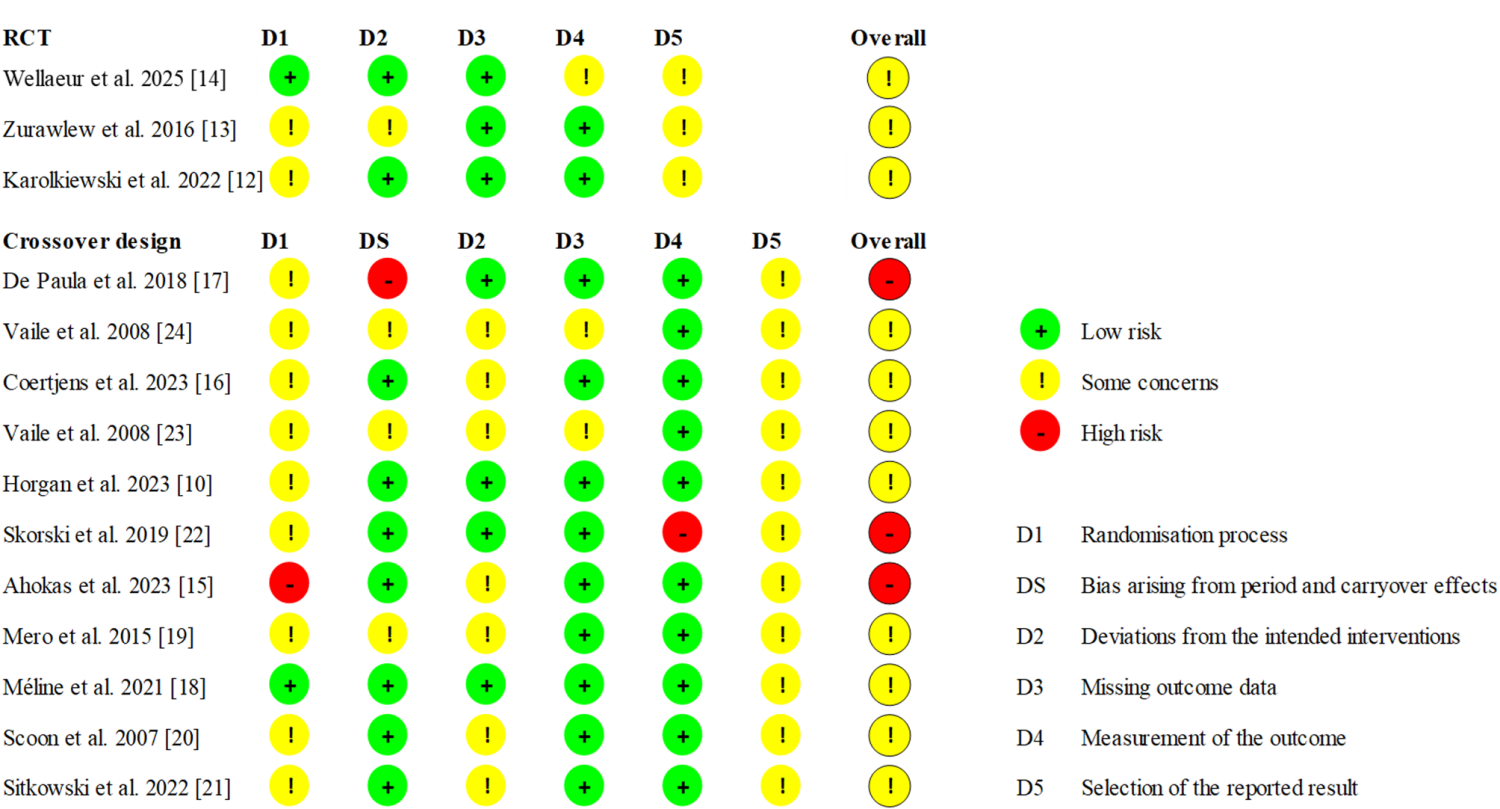
Individual ratings for randomised controlled trials and crossover designs from the Risk of Bias 2 analyses. *RCT* randomised controlled trial, *SPOLIT* SportLiteraturdatenbank

A total of three studies were rated as having ‘high risk of bias’, and eleven studies were rated as having ‘some concerns’ (Fig. 2). The most common sources of bias were in the randomisation process and the selection of reported results. The risks were attributed to missing information related to the randomisation process and missing data. In addition, only one study was prospectively registered, and statistical analyses plans were not available.

## Discussion

According to the results extracted from the studies included in this systematic review, the effects of post-exercise whole-body heat exposure on acute recovery and training adaptations present uncertain outcomes. In four of the nine studies [15, 16, 19, 23] examining acute recovery, some positive heat exposure-induced effects on physical performance were found. However, enhanced performance during acute recovery was not consistently observed across various performance variables. Only one study on acute recovery of physical performance reported negative effects [22]. In two [13, 20] out of five studies examining training adaptations, beneficial effects of repeated use of heat exposure after exercise were observed, although in one study some negative effects were found [18].

Contradictory findings were observed with respect to acute recovery of endurance performance and the application of post-exercise heat. Whilst HWI had beneficial effects on immediate cycling performance in the Wingate test after immersion [16] and some beneficial effects on average power during a cycling time trial over a five day training period [24], HWI did not improve running time trial performance after five hours of recovery compared to passive recovery [17]. Coertjens et al. [16] postulated that improved anaerobic performance in the Wingate test immediately after HWI could be explained by increased muscle temperature, which enhanced restoration of energy stores and function of the nervous system, including increased corticospinal excitability, decreased intracortical inhibition, and increased intracortical facilitation. Contrary to the authors’ hypothesis that HWI would enhance lactate clearance due to vasodilation and hydrostatic pressure-induced fluid shifts toward central blood volume, blood lactate concentration remained similar between HWI and passive recovery over the 10-minute exposure period [16]. However, the authors speculated that a longer heat exposure could have revealed differences in lactate levels between HWI and passive recovery [16], which could happen possibly due to the greater and longer increase in body temperature. Indeed, a 30 min post-exercise IRS (45 °C) after submaximal running session was found to enhance lactate clearance [26]. Furthermore, HWI performed immediately post-exercise attenuated the recovery of ventilation and heart rate (HR) [16]. The increase in ventilation observed during HWI might be due to the body’s need to facilitate heat dissipation, as the absorbed energy during HWI may impede the release of cellular heat energy generated during physical exercise [16]. In addition, higher HR was found during HWI compared to passive recovery when it was performed after eccentric strength exercise and a 90 min running session [17]. However, there were no differences in HR 0–30 min after the immersion and during a running time trial five hours later, and rectal temperature (T_rec_) was not statistically higher during the HWI and 0–30 min after that compared to passive recovery [17]. Furthermore, nocturnal HR and heart rate variability (HRV) did not differ between IRS and passive recovery after a resistance exercise session, even though HR was higher and HRV lower during heat exposure in IRS [15]. Thus, it seems that parasympathetic reactivation occurs soon after heating. Vaile et al. [24] also found higher T_rec_ immediately- and 15 min post heat exposure, but there was no difference in T_rec_ the following day. Therefore, it appears that the transient physiological stress reflected by elevated HR and T_rec_ during HWI had no effect on endurance performance, as T_rec_ typically returns to baseline within the first 30 min after heat exposure, and HR follows shortly after, according to both the studies included in this review and additional literature on physiological responses to heat exposures [17, 24, 27, 28], with the latter studies [27, 28] excluded from the review due to incompatible study designs.

While some positive effects on endurance performance were observed, post-exercise traditional sauna bathing impaired swimming performance compared to placebo, in which participants applied massage oil to their body while passively resting [22]. Compared to placebo, sauna bathing did not however affect blood lactate concentration and HR during the swimming performance, and there was no change in hematocrit values [22]. It remains unclear why performance declined in this study [22], as these findings conflict with other studies that have reported either no negative effects [17] or even benefits [16, 24] of post-exercise heat exposure on endurance performance. One explanation could be the time course of the performance measures, as elevated body temperature, such as that induced by a warm-up, is known to enhance power production [29], which could partly explain the improved mean power output observed immediately after HWI in the study by Coertjens et al. [16]. In contrast, when performance was measured the following day, the effect of prior heat exposure was generally minimal [17, 24] or even negative [22], suggesting that the short-term benefits of elevated body temperature do not persist. Alternatively, it has been proposed that the thermal stress induced by traditional sauna bathing could have caused the negative effects on swimming performance [22]. Despite the fact that the sauna had a higher temperature and longer duration (80–85 °C, 3 × 8 min) compared to HWI (38–40 °C, 10–15 min), thermal conductivity is higher in hot water than in hot air [30]. This makes it challenging to compare the heat strain experienced by participants to different heat exposure methods. Another explanation for the varying findings regarding acute recovery of performance is the possible difference in total training load before and during the study periods, which has not been described [16, 17, 22, 24]. Additionally, the participants in Skorski et al. [22] were young competitive athletes, who were not experienced with sauna bathing. Thus, it is possible that the combination of heat exposure and the overall training load was more stressful on the young athletes’ bodies.

A beneficial effect of heat was found in one of the five studies that investigated the acute recovery of isometric strength production [23]. A differentiating factor in that study was that participants engaged in HWI four times during the 72-h recovery period, which means that the heat dose was higher compared to a single heat exposure, and repeated use during the recovery period might maintain or reactivate physiological adaptations. In the remaining four studies, IRS [15, 19] and HWI [10, 14] were not found to improve recovery of isometric strength production compared to the control condition but in each case, the recovery method was used only once or twice on the same day as the exercise. Although the only study reporting beneficial effects applied the heat exposure method multiple times throughout the recovery period, improved recovery was already found 24 h after a single HWI [23]. Another reason for the varying results could therefore be differences in the loading protocols. Vaile et al. [23] used high intensity eccentric resistance loading (100–120% 1RM), whereas other studies [10, 15, 19] used hypertrophic or complex resistance training at lower intensities (75–95% 1RM). Thus, the loading used in the study by Vaile et al. [23] might have caused greater muscle damage, making recovery procedures potentially more beneficial.

In three studies, recovery of lower body power assessed by CMJ was improved after HWI [10] and IRS [15, 19], and in one study there were no differences in recovery of CMJ height between traditional sauna and placebo [22]. It should also be noted that only a treatment effect (no interaction effect) was found after HWI [10] and improved recovery of CMJ height was observed only after endurance exercise loading, not after hypertrophic loading [19]. Furthermore, recovery of 20-m sprint [15], SJ [10], and loaded SJ [23] performance was not influenced by heat exposure. Interestingly, the positive effect of heat exposure on CMJ was found from immediately after heat exposure until 38 h later [10, 15, 19]. It therefore appears that heat could have a positive impact on the recovery of power production. The inconsistency between the findings regarding the recovery of jumping performance and MVC may be explained by the differing neuromuscular demands of the stretch-shortening cycle [31] or by enhanced recovery of fast-twitch muscle fibres [32].

Previous studies investigating the effects of heat on recovery of strength and power capacities also assessed some physiological factors that might explain changes in performance capacity, such as indirect markers of muscle damage (creatine kinase (CK), myoglobin, and lactic acid dehydrogenase) and inflammation (interleukin-6 (IL-6) and other cytokines [10, 15, 23]. There were no differences between CK activity and myoglobin concentration between IRS and passive recovery [15], and HWI was not found to influence CK activity and myoglobin, lactic acid dehydrogenase, and IL-6 concentrations [14, 23]. Nevertheless, the only study where a positive effect on the recovery of maximal strength production was found also observed decreased CK activity 48 h post-exercise in the HWI-condition compared to passive recovery [23]. Furthermore, a study based on the same design and participants as Horgan et al. [10] found lower CK activity in HWI compared to control [33], but no differences were found in myoglobin, pro-inflammatory, and anti-inflammatory cytokines between HWI and control [33]. Collectively, these results demonstrate that the use of post-exercise heat methods had minimal or non-existent effect on indirect muscle damage markers and inflammatory responses. However, local heat therapy has been found to attenuate the intramuscular inflammatory response [34] and expedite inflammation after injury in animal studies [35]. McGorm et al. [6] speculated that on this basis, heat could enhance the recovery process by affecting inflammation [6]. Other possible mechanisms governing the potential positive effects of heating on muscle repair are heat shock proteins (HSPs) [6, 36]. However, muscle temperature plays a crucial role in upregulating the expression of HSP [6, 36], and it has been speculated that a muscle temperature elevation to 38 °C [37] or even 40 °C [6] is necessary to augment beneficial responses in muscle repair. None of the included studies addressing acute recovery of physical performance measured intramuscular temperature, which would be expected for studies intending to elucidate physiological mechanisms underpinning the effect of heat application on recovery.

Previous meta-analytical data have revealed that post-exercise heat can relieve delayed onset muscle soreness [38]. However, only local heat was found to be effective, rather than whole-body heat methods [38]. In the present review, subjective muscle soreness was studied in only four [10, 14, 15, 23] out of eight studies investigating the effects of heat methods on recovery. Only one of those three studies observed that heat relieved muscle soreness compared to passive recovery [15]. However, other benefits on subjective measures of recovery were found, such as increased subjective sleep quality [10] and perceived recovery [15], and it seems that psychological effects are linked to the athletes’ mood and work attitude and can be of major importance for performance [39, 40]. It should be noted that these study protocols did not include placebo treatment and athletes’ beliefs might have affected the results. The only study including placebo treatment in this systematic review reported higher overall stress among participants on the morning following a traditional sauna compared to placebo treatment, but no differences in perceived recovery and performance capability [22].

Repeated use of post-exercise heat was found to improve running performance [20], at least in hot conditions [13]. In these studies, physiological changes related to heat acclimation were also found. Total blood volume and plasma volume were higher after a sauna intervention compared to a control intervention, and positively correlated with running performance [20]. Furthermore, endurance training with HWI increased plasma volume and lowered T_rec_, end of exercise T_rec_ and heart rate (HR) when no changes were found in the control group [13]. Repeated use of post-exercise heat was not found to further improve VO_2max_ or VO_2peak_ in a graded cycle ergometry test [12, 18, 21]. Physiological variables related to heat acclimation were included only in one of those studies, where no differences were found in total hemoglobin mass, red cell volume, blood volume, plasma volume, and erythropoietin between sauna and training alone [21]. It should also be noted that Karolkiewicz et al. [12] and Sitkowski et al. [21] may have used some of the same participants and protocols, but different study designs. In addition, the participants were allowed to take a cold shower during the three-minute breaks between sauna bathing periods, which might have decreased the body temperature and thus other physiological responses.

The impact of repeated use of post-exercise HWI on strength, power and speed training adaptations was explored in one study, which produced conflicting results [18]. In a study of six elite speed-skaters, a HWI intervention elicited larger increases in maximal isometric strength of the knee extensors compared to passive recovery [18]. Despite this, the same group experienced larger increases in cross sectional area of the knee extensor and flexor muscles in the passive recovery intervention, alongside an improvement in 1.5-lap speed-skating performance [18]. It is important to acknowledge that these findings are from only six participants, even though they were elite athletes.

Local heat exposure has also been found to enhance muscle hypertrophy [7, 41], which was not observed in the study investigating speed-skating performance and HWI [18]. Studies where local heat was found to improve muscle hypertrophy used heat exposure only before [42] or before and during exercise sessions [43], long-term passive heat [7], and animal subjects [41]. Training in a heated environment [44, 45], local pre-heating [42, 43] and long-term passive heating [7–9] have also been found to improve maximal force production. It should be noted that the participants in these studies were either physically inactive [7, 8, 43, 44] or recreationally active but did not participate in any resistance training [9, 42]. It is thought that trained athletes’ adaptations to training may be reduced as compared to these groups due to pre-existing exercise-induced adaptations [10]. A potential mechanism for enhanced muscular function and hypertrophy could be activation of HSPs [6], as they protect cells from damage and improve muscle regeneration [46]. As previously noted, muscle temperature plays an important role in upregulating the expression of HSPs [6, 36], and it might be that intramuscular temperature, which was not measured, was not high enough in the study by Méline et al. [18]. In another study, a 10-minute post-exercise HWI (40 °C) was found to elevate muscle temperature compared to baseline and passive recovery [47]. Nevertheless, the intramuscular temperature was only 37.2 (0.3) °C at a depth of 3 cm immediately after the HWI. Méline et al. [18] used the same water temperature, but the duration of the immersion was 20 min. Thus, the intramuscular temperature might have been closer to 38 °C. Another possible mechanism behind the positive effects of heating involves mammalian target of rapamycin (mTOR) kinases [6], which stimulate cell growth [48]. Heating might also alter gene expression, particularly in relation to muscle hypertrophy and atrophy [49].

Based on the current research data, there are conflicting results about the benefits of post-exercise whole-body heat exposures, and the physiological mechanisms behind the possible benefits are at the level of speculation because of the diverse research methodology used in the studies and the missing information about the physiological responses. Future studies should include body temperature measurements, as this would help to understand the reasons and the mechanisms behind the possible beneficial effects, and compare different heating methods. Traditional saunas and HWI heat the occupant by convection of the heated air or water, but as mentioned above, thermal conductivity is higher in hot water than in heated air [30]. Instead of through convection, IRS radiates heat. Hydrostatic pressure also affects the physiological response to HWI. This results from displacement of peripheral fluid toward the central blood volume, generating greater stroke volume, and cardiac output [50, 51]. Thus, comparing heating methods is not possible without measuring physiological responses, like body temperature.

Female participants were included in 3 of the 14 studies included this review. It has been observed that medium term heat acclimation period affects both women and men in similar ways [52], while short-term heat acclimation might have a reduced effect in women compared to men [53]. Furthermore, physiological responses to endurance [54, 55] and strength [56, 57] training differ between sexes and so more female participants should be included in studies of this type. However, thermoregulatory changes due to the menstrual cycle phase and hormonal contraceptive use can affect heat responses [58]. To reduce hormonal impact, testing eumenorrheic participants during the early follicular phase is recommended, along with collecting menstrual cycle data for analysis [59]. Additionally, it should be noted that the number of participants in the studies included in this review was small, which may have reduced the likelihood of detecting clear effects.

## Conclusion

Based on the current research data, no definitive consensus can be drawn regarding the effects of post-exercise whole-body heating on acute recovery or training adaptations. It seems that improved physical performance is most likely to be seen immediately after exposure to heat. Furthermore, repeated use of post-exercise heat exposure might be beneficial for endurance performance if physiological responses related to heat acclimation have occurred [13, 20]. To gain a comprehensive understanding of different post-exercise heating methods and their benefits, future studies should include measuring physiological responses such as body temperature, in addition to physical performance. It should also be noted that whole-body heat exposures could add additional stress to an athlete’s total load and recovery regime [58], which may have translated to a negative effect on physical performance in one study [22]. Thus, the objectives and timing of post-exercise heat interventions should be carefully considered.

## Data Availability

Data will be made available on reasonable request.

## Abbreviations

HWI: Hot water immersion
PRISMA: Preferred Reporting Items for Systematic Reviews and Meta-Analysis
IRS: Infrared sauna
CMJ: Counter-movement jump
SJ: Squat jump
TWI: Thermoneutral water immersion
VO_2max_: Maximal oxygen uptake
VO_2peak_: Peak oxygen uptake
HR: Heart rate
T_rec_: Rectal temperature
HRV: Heart rate variability
CK: Creatine kinase
IL-6: Interleukin-6
HSP: Heat shock protein
mTOR: Mammalian target of rapamycin
